# Dignity-Preserving Dementia Care in Old Age Homes in Nepal: Healthcare Professionals’ Perspectives

**DOI:** 10.1177/23333936251369444

**Published:** 2025-08-27

**Authors:** Oscar Tranvåg, Soni Shrestha

**Affiliations:** 1 Western Norway University of Applied Sciences, Bergen, Norway; 2 Oslo University Hospital, Norway; 3 University of Bergen, Norway

**Keywords:** dementia, dementia care, dignity, healthcare professional, old age homes, Nepal

## Abstract

In recent decades, there has been growing interest in research on dignity in care for persons with dementia living in high-income countries. In contrast, such research remains limited in low- and middle-income countries. This study aimed to explore healthcare professionals’ perceptions of the critical qualities inherent in dignity-preserving care for persons with dementia living in old age homes in Nepal. Using a hermeneutical approach, we conducted qualitative in-depth interviews with eleven healthcare professionals. Our analysis revealed that participants’ understanding of dignity-preserving care was rooted in their ontological and cultural belief of ‘Acknowledging the equal worth of each human being’. Grounded in this foundational belief, critical elements of dignity-preserving care practices focused on ‘sheltering the self-esteem and promoting a meaningful everyday life of persons with dementia’. These practices involved ‘being respectful and protecting the self-identity of each person’; ‘recognising the need for being a person who people listen to’; ‘showing regard for each person’s efforts and contributions in life’; ‘safeguarding the person’s sense of belonging’, and; ‘facilitating personally valued activities’, and were considered as critical qualities of dignity-preserving care. The study provides valuable insights for improving care strategies and informing policy developments for dignity-preserving care for persons with dementia.

## Introduction

The World Health Organization (2023) describes dementia as a syndrome characterised by progressive and irreversible cognitive decline that affects multiple areas of functions, including memory, communication skills, reasoning and the ability to carry out daily activities. Persons with dementia may also develop associated behavioural and psychological symptoms, also recognised as neuropsychiatric symptoms that manifest along with the syndrome of dementia, such as hallucinations, apathy, delusions, disinhibition, anxiety or depression. These symptoms include perceptual, emotional, and behavioural disturbances that are similar to those exhibited in psychiatric disorders (Cloak et al., 2024). Worldwide, more than 55 million people are living with dementia – a number which is estimated to increase to 78 million by 2030 and 139 million by 2050 (World Health Organization, 2021).

As dementia progresses, the need for nursing care and assistance for the activities of daily living increases (Van der Geugten & Goossensen, 2020). In this process, persons living with dementia need more support to be able to interact with the environment, and they are at risk of alteration in dignity (Torossian, 2021). In recent decades, there has been increased interest in the dignity of persons with dementia which has led to expanding our understanding and conceptualisation of what dignity entails for this particular group of people (Tranvåg et al., 2013). Similarly, there are studies investigating how dignity in nursing practice is understood in various care settings. For example, in their systematic review Lindwall and Lohne (2021) explored how human dignity is described in caring science research, aiming to gain a broader understanding of differences and similarities across caring contexts such as from acute, psychiatric, elderly and rehabilitation care. In another study, Franco et al. (2021) conducted a synthesis review on dignity in nursing care to identify key attributes of dignity, and discuss antecedents and consequences to clarify the substance of dignity, with implications for nursing education and clinical practice. Additionally, there has been rising interest in conducting qualitative research to explore and increase the understanding of dignity in care for persons with dementia – from the perspectives of healthcare professionals (HCPs). Our review of the literature, described below, shows that most of these studies are performed in high-income Western countries such as the UK, Sweden, Norway, Australia and Canada.

### Previous Research

In their study conducted in the UK, Manthorpe et al. (2010) found that HCPs perceived that dignity-preserving care contained several aspects. Respecting the person’s need to feel in control and by doing so also maintaining the person’s trust and confidence was addressed as vital. Finding and retaining the person’s sense of status in their own eyes, and offering person-centred support were also perceived as crucial dignity-preserving aspects. The Swedish study by Zingmark et al. (2002) shows that HCPs emphasised being observant of the personality of each person, while confirming and preserving their sense of self and experience of being of value. Discretely offering help and sheltering the person from disrespectful treatment were also found as critical in dignity-preserving dementia care. In the review of previous research, three other Swedish studies should be mentioned. First, Randers and Mattiasson (2004) found that empathising with the person with dementia, enhancing the person’s resources with respect to social interaction, and reminiscing glimpses of the person’s life history were perceived as dignifying by HCPs. This was also the case in confirming the person’s authentic autonomy and underpinning negative and positive autonomy with respect for the individual’s integrity as a state of wholeness. Second, Sävenstedt et al. (2006) documented that HCPs perceived information- and communication technology applications as dignifying when promoting the person’s security, freedom and social interactions – opposite to information- and communication technology as unworthy surveillance. Third, Örulv and Nikku (2007) found that in dignity-threatening conflicts between persons with dementia, HCPs emphasised choosing an appropriate dignity-preserving way of handling these by non-interference, wait-and-see, forestalling or immediate interference. Thereafter, a fitting strategy was carried out choosing between direct confrontation, avoiding–confirming strategy, separating the persons with dementia, confirming–justifying strategy or encouraging ‘good conduct’.

The study of Borbasi et al. (2006) conducted in Australia, showed that HCPs emphasised that creating a purpose-built environment and having time to engage in conversations with the person living with dementia were vital for dignity in care. Being knowledgeable, flexible and willing to give the extra effort required for their care were also found to be core dimensions in dignifying care. In another Australian study, Rodriquez (2011) found that emphasizing emotional attachment was perceived as a resource that promoted the experience of dignity for persons with dementia in nursing home, as well as for HCPs themselves – treating the person as a dignified individual worthy of respect and care. Moreover, Rodriquez (2009) found that recognizing when verbal and physical violence are illness-related and unintentional, and enduring such incidents without anger or regret, were considered vital aspects of dignity-preserving care.

The study of Manthorpe et al. (2010), conducted in the UK, found that HCPs considered both the person’s needs as well as rights, emphasizing the importance of balancing individual choice with those needs and the duty of care as essential elements of dignity-preserving care for persons living with dementia. In Norway, Jakobsen and Sørlie (2010) found that HCPs emphasised going out for walks in order to decrease the experience of lost freedom due to locked doors for persons with dementia as a vital dignity-preserving care measure. They also found that, in certain situations, HCPs considered the use of mild restraint to be dignity-preserving when the person with dementia was unable to make informed decisions about their own needs. Finding the right time for using persuasion was identified as critical to preserve the person’s self-determination. In another Norwegian study, Rognstad and Nåden (2011) reported that, in challenging situations, when HCP questioned the ability of persons with dementia to make sound autonomous choices, they emphasised finding a dignity-preserving balance respecting the person’s right to autonomy against the necessity of making decisions on behalf of the person with dementia. In some situations, this involved using a certain degree of mild restraint in accordance with the given restraining order, but accomplishing it in a manner which is as forbearing as possible, while acting in calm and trusting way to prevent agitation and aggression. A Norwegian meta-synthesis conducted by Tranvåg et al. (2013) exploring HCPs’ perception and practise of dignity-preserving dementia care found that balancing individual choices among persons who were no longer able to make sound decisions against the duty of making choices on behalf of the person, involving persuasion and/or mild restraint, was considered as a crucial dignifying aspect in certain situations. Moreover, they found that advocating the person’s autonomy and integrity as a vital foundation, involving having compassion for the person, confirming the person’s worthiness and sense of self as well as creating a humane and purposeful environment were useful. On this basis, ‘Sheltering human worth – remembering those who forget’ was identified as a core value and comprehensive motive in dignity-preserving dementia care.

In Canada, Chochinov et al. (2005) have developed a Dignity therapy, which is an intervention wherein trained HCPs take the patient through a guided interview to record significant life events and other information that the person would like others to know about them. Originally, this approach was designed to promote the sense of meaning and purpose for persons nearing the end of life. Johnston et al. (2016) in the UK have reported that Dignity Therapy, conducted by HCPs, is found to be feasible and potentially beneficial in person-centred care and the promotion of quality of life among persons in early stages of dementia as well.

While the body of knowledge on this topic is expanding in high-income Western countries, research addressing this subject in low- and middle-income countries remains scarce. There is limited understanding of whether HCPs in these settings ground their respect for the dignity of persons living with dementia in the same values and motivations as their counterparts in high-income countries. A recent study conducted in Israel (Kaplan & Bentwich, 2023) reports dissimilarities in the actual behaviour of HCPs, suggesting that cross-cultural differences may exist in how dignity is respected for persons with dementia in nursing homes. This highlights the need to develop research-based knowledge on the critical qualities that constitutes dignity-preserving care for persons with dementia, particularly within the context of low- and middle-income countries.

### Nepalese Context

Nepal is a lower-middle-income country in South Asia (The World Bank, 2022), in which people aged 60 and over are considered as senior citizens under the Senior Citizens Act (Nepal Law Commission, 2006). The national Central Bureau of Statistics (2021) reported that 2,977,318 senior citizens were living in Nepal in 2021, marking a growth rate of 38.2% in comparison to the census of 2011 which reported 2,154,003 senior citizens. With this rise in the number of senior citizens, the prevalence of dementia is also increasing, as age is the primary risk factor for this syndrome (Hou et al., 2019). A study conducted in 2013 estimated that approximately 135,000 people were living with dementia in Nepal, and that this number was expected to double every 20 years – mirroring the trends observed in Western countries (Jha & Sapkota, 2013).

In Nepal, cognitive impairment screening is provided by Medical college hospitals and some corporate hospitals, including zonal hospitals (Nepal & Sapkota, 2017). Only hospital-based data are currently available on the prevalence of dementia. These show that 11.4% of individuals attending psychiatric clinics in tertiary care centres, such as central hospitals and regional hospital, over the course of 1 year were diagnosed with dementia (Nepal & Sapkota, 2017). A study published in 2019 revealed that no nursing homes specifically designated for people with dementia had been established in Nepal, although some private care homes did exist (Sapkota & Subedi, 2019).

Nepal has a long established culture of respecting senior citizens (Acharya, 2008). The majority of them live with their families and usually with their sons (Parker et al., 2014). However, the shift from joint to nuclear family structures (Acharya, 2008), the migration of children to urban areas or abroad in search for better opportunities (Dhital et al., 2015) along with urbanisation, modern values, and global integration, have led to societal changes that have weakened traditional social values and structures (M. Shrestha et al., 2019). These aspects are contributing to a shift in the way care is provided to senior citizens (Dhital et al., 2015). Vulnerable to mental health problems like depression, loneliness and other mental diseases (Chalise, 2010, 2012, 2014), many seniors are often left to live on their own (Chalise & Shrestha, 2005) or to move to institutions like old age homes (OAHs) (Akbar et al., 2018; Tawiah, 2011). Originally, OAHs in Nepal were established by the government to care for elderly persons who had no children or families to support them. These homes were often situated near religious sites, reflecting cultural values and offering spiritual solace to the residents (Chalise, 2014). In contrast, nursing homes are profit-based institutions that are typically smaller than hospitals. They provide a wide range of clinical care services, including outpatient departments, intensive care units, operation theatre wards, general wards, emergency wards, medical wards, gynaecological wards, orthopaedic wards, paediatric wards, and psychiatric wards (Pathak & Gaire, 2020). In recent decades, there has been a noticeable increase in the number of senior citizens residing in OAHs, accompanied by a rise in the number of such facilities, particularly in urban areas (Rai et al., 2018). A study conducted across twelve public and private OAHs (in that study referred to as geriatric homes) in Kathmandu revealed a high prevalence of dementia, affecting more than two-thirds (75.65%) of the residents (Singh et al., 2021). Previous studies also show that OAHs’ residents have an increased risk of having mental health problems like depression and loneliness (Chalise, 2014; Mali et al., 2021). Insufficient proficiency in managing residents’ cognitive disturbances and behavioural and psychological symptoms in dementia, along with limited educational training of HCPs and inadequate resources to ensure sufficient numbers of HCPs and medical doctors for proper diagnostic examination, treatment and dementia-specific care have been identified as critical challenges to offering quality dementia care in the setting of OAHs in Nepal (Shrestha & Tranvåg, 2022). A study conducted in the OAHs in Northern India, a neighbouring country to Nepal, highlights that residents of OAHs often face daily challenges stemming from a highly institutionalised, depersonalised, and bureaucratic environment. Within this setting, they endure rigid schedules, separation from family, isolation from their natural social networks, anxiety related to adopting to a new environment, and frequent exposure to illness and death among fellow residents (Akbar et al., 2018).

Currently, there is limited knowledge on how the dignity of persons with dementia residing in Nepalese OAHs can be preserved, as this topic, to our knowledge, has not been previously explored in depth. Gaining a deeper understanding of this issue could significantly inform healthcare practices and policy development in Nepal, particularly in addressing the needs of this vulnerable population. Investigating the perspectives of Nepalese HCPs who are directly involved in the daily care of persons with dementia in OAHs can offer valuable insights into the essential qualities of dignity-preserving care. Such knowledge would serve as a critical evidence base for designing and implementing research-informed, dignity-preserving dementia care practices within Nepalese OAHs.

The aim of this study was to explore and describe the critical qualities inherent in dignity-preserving care for persons with dementia living in OAHs in Nepal, as perceived by the HCPs responsible for their daily care.

## Methods

### Research Design

The five step hermeneutical method by V. Fleming et al. (2003), which is based on the hermeneutical epistemology of Gadamer (2004), was chosen as this can be employed in nursing research in order to articulate the process of interpreting and understanding people’s experiences and perceptions (V. Fleming et al., 2003; Phillips, 2007). The initial step in this exploratory process was *deciding upon the research question*, which is an essential step in relation to underpinning the methodology of the study. To ensure internal study consistency, and to expand the knowledge in the field of nursing, we had focused our attention on developing a precise research question that has previously been sparsely explored. In the second step, we discussed the research question and reflected upon our own preformed knowledge and beforehand understanding. This allowed us to *identify our own pre-understanding* of what we might find when exploring HCPs’ perceptions of dignity-preserving dementia care in Nepalese OAHs. The pre-understanding will be described in the following section. The last three steps in the hermeneutical process: *gaining understanding through dialogue with participant; gaining understanding through dialogue with text*, and; *establishing trustworthiness* (V. Fleming et al., 2003) will thereafter be illuminated.

### Pre-understanding

When conducting research grounded in Gadamer’s philosophical hermeneutics, it is essential for researchers to identify and reflect upon their pre-understandings of the subject matter. This reflexive process enables them to move beyond their initial assumptions, allowing for a deeper understanding of the phenomenon and the possibility of transcending their own ‘horizon’ (V. Fleming et al., 2003, p. 117). These pre-understandings should be described and discussed in the research report (V. Fleming et al., 2003).

In this study, both researchers, OT (a mental health nurse from Norway), and SS (a medical doctor from Nepal), respectively, had professional experience in caring for older people with dementia. The choice of Nepal as the study setting aligns with the researchers’ concern for global health challenges and the need for more research in the Nepalese context. Our pre-understanding was based on the belief that HCPs in Nepalese OAHs perceived dignity as an essential quality of every human being and that dignity-preserving care for persons with dementia unfolds in their everyday relational interactions in the form of respect and honour. How HCPs themselves would describe critical qualities constituting dignity-preserving care within this context was, however, unknown to us.

### Participants and Recruitment

The study was conducted in one governmental, three private and one semi-private OAHs in Kathmandu. These OAHs were selected based on publicly available information from their websites, which indicated that the three private OAHs housed individuals with dementia. Additionally, the government and semi-private OAHs were included based on our understanding that they accommodate a high number of older residents. First, a request for permission to conduct the study at the respective OAHs, including recruiting participants among their HCPs, was directed to, and granted by the manager at each OAH. Thereafter, a purposeful sampling strategy was used to select HCPs with relevant experience in caring for persons with dementia. With the support of managers from each OAH, we intentionally selected potential participants based on the following inclusion criteria: has formal education as an HCP and at least 6 months of experience in the daily care of OAH persons with dementia. This approach ensured the inclusion of participants with firsthand experience in dementia care. A total of eleven HCPs, nine nurses and two physiotherapists, gave their written consent to participate, among which there were two nurses from the government OAH, one nurse from the semi-private OAH, six nurses and two physiotherapists from the three private OAHs as detailed in Table 1. The HCPs held one of the following educational qualifications: Bachelor’s degree in nursing, Auxiliary Nursing Midwife (ANM), Bachelor’s and Master’s degree in Physiotherapy and Proficiency Certificate Level (PCL) Nursing. PCL nursing is a 3 years academic course; equivalent to diploma and the graduates can work as staff nurses and also can pursue Bachelor of Science in nursing (Nepal Institute of Health Sciences, 2020). While the nurses were directly involved in assessing health conditions, providing medicines and following up on doctors’ order on a daily basis, the physiotherapists had focused their attention on the management of persons with dementia’s functional problems and care needs, such as providing exercise therapy based on their individual ability and mobility status. Physiotherapy services were only available in the private OAHs, whereas in other OAHs, nurses and other staff provided simpler forms of exercises.

**Table 1.** Details of Participants.

Participants	Age	Sex	Profession	Education
1	25–30	Female	Nurse	Proficiency Certificate Level (PCL) Nursing
2	50–55	Female	Nurse	Proficiency Certificate Level (PCL) Nursing
3	40–45	Female	Nurse	Auxiliary Nursing Midwife (ANM)
4	30–35	Female	Nurse and medical counsellor	Bachelor in Nursing Hospitality and Management
5	25–30	Female	Nurse	Proficiency Certificate Level (PCL) Nursing, Bachelor in Nursing
6	25–30	Female	Physiotherapist	Bachelor and Master in Physiotherapy
7	20–25	Female	Nurse	Proficiency Certificate Level (PCL) Nursing
8	20–25	Female	Nurse	Proficiency Certificate Level (PCL) Nursing
9	30–35	Male	Physiotherapist	Bachelor and Master in Physiotherapy
10	30–35	Female	Charge Nurse	Proficiency Certificate Level (PCL) Nursing, Bachelor in Nursing
11	20–25	Female	Nurse	Proficiency Certificate Level (PCL) Nursing

### Data Collection

In the third step of the hermeneutical method, we focused on gaining understanding through dialogue with participants by speaking with them and being open to their experiences and opinions, as it is through dialogues understanding becomes possible (V. Fleming et al., 2003). We emphasised conducting the dialogues in a language that was understandable for both parties and focused on clarifying linguistic ambiguities when they arose. In this process, emphasis was put on not attempting to understand the matter of interest through the eyes of the participants, but instead working together with the participants with the aim of reaching a shared understanding (V. Fleming et al., 2003).

The dialogues took place within the OAH facilities and were conducted by SS. Consistent with hermeneutic dialogues (Vandermause & Fleming, 2011), we emphasised that our questions should be a starting point allowing the participants to think along and articulate their experiences and perspectives. Based on the participants’ response, follow-up questions were asked to further elaborate and clarify their experiences and perspectives. Examples of our main questions that guided the dialogues were: How do you understand the term ‘dignity’? Can you tell me how the dignity of persons with dementia can be preserved, as you perceive this from your professional perspective? We emphasised being open for the horizon of the other, whilst being explorative, engaged and conscious of own pre-understandings in the dialogue with each participant to lay foundation for a fusion of horizons – a shared understanding (V. Fleming et al., 2003) of the participants’ perception of dignity-preserving care for residents with dementia.

One dialogue was first conducted with each of the eleven participants. Four participants also gave their consent to take part in a second dialogue. These follow-up interviews were conducted to deepen our understanding of the findings and to validate the preliminary interpretations derived from the initial interviews with the eleven participants. Additionally, these interviews allowed us to explore new insights that emerged through our initial analysis. The scheduled time for all the dialogues were 60 to 90 min, but some of them lasted shorter as the data collection was conducted during the participants’ working hours and some of them had to return to their post earlier than expected. All dialogues were conducted in the Nepali language by researcher SS, audio-recorded and transcribed verbatim in English by SS who has a deep understanding of both languages, enabling her to capture the content, tone, and meaning in the dialogues – making them understandable and interpretable also for the English-speaking researcher OT.

### Data Analysis and Interpretation

Entering the fourth step of V. Fleming et al. (2003) hermeneutical method, we put an emphasis on *gaining understanding through dialogue with the text*. The initial analysis was conducted after the first series of dialogues, noting keywords and writing initial summaries (V. Fleming et al., 2003). We then proceeded with the next dialogues in which preliminary understanding of the first dialogues were further explored in the dialogue with new participants. In this process, several individual readings of each transcribed dialogue were performed to gain further understanding of the parts of the text and the text as a whole, having joint meetings in which we shared and discussed our preliminary interpretations of each transcribed dialogue text – and all dialogue texts as a whole. Throughout this hermeneutical circular process, we went back and forth in each transcribed dialogue text to explore the parts and the text as a whole. This made it possible for us to investigate other possible interpretations, as parts and as a whole, so as to let the substance come forward and prevent our pre-understanding from concealing its essence. All texts were examined to find an expression reflecting the core meaning of the text as whole. To be able to develop themes that could describe the meaning of the text, each formulation was thoroughly explored before relating its meaning to the meaning of the text as a whole. In this process, we had focused attention on identifying passages we found to be representative for the shared understanding of the study participants and us as researchers (V. Fleming et al., 2003).

The fifth and final step in this hermeneutic method reminds us of the importance of *establishing trustworthiness* (V. Fleming et al., 2003) throughout the research process. In addition, we used the five criteria of research credibility, dependability, confirmability, transferability and authenticity (Guba & Lincoln, 1994; Lincoln & Guba, 1985) as a guiding lens. To enhance study credibility, we wrote reflexive journaling throughout the process of planning, data collection and data analysis. We maintained dependability through emphasising the transparent documentation of the research process. We emphasised authenticity by providing rich empirical data to the readers that genuinely reflect the participants own articulated perspectives and experiences. We heightened study confirmability by demonstrating that the interpretations and findings were clearly derived from the information shared by the HCPs – while simultaneously being aware of the possibility of interpretive bias, thus reflecting upon our pre-understandings and questioning our initial interpretations while searching for other possible interpretations of the empirical data. To increase study transferability, we emphasised providing thick descriptions of how we conducted the research process as well as of the contexts of Nepalese OAHs in which the study was carried out in order to help readers assess whether our findings may be transferable to other contexts, situations and populations.

### Ethical Considerations

The study was approved by the Nepal Health Research Council (Approval Reg. No. 215/2018) and was conducted in accordance with the ethics guidelines for research ethics formulated in the Declaration of Helsinki (World Medical Association, 2025). Permission for conducting interviews was sought separately from each OAH. Both verbal and written information about the study was provided to the manager of each OAH, and approval was received for the study to be performed. All the participants received verbal and written information about the study, after which informed consent was obtained from each of them. All the participants were informed about their rights to withdraw from the study without any consequences and without stating any reason. To ensure confidential handling of data, the audio recording of the interview, transcriptions, and contact information were securely stored and processed through a data platform with multi-factor authentication. Personally identifiable information such as names and place names were omitted from the transcriptions. Contact information was replaced with a code that was stored in a separate document so that it could not be linked to the interview data. After transcription, the audio recordings and contact information were deleted.

## Results

**Figure 1.**
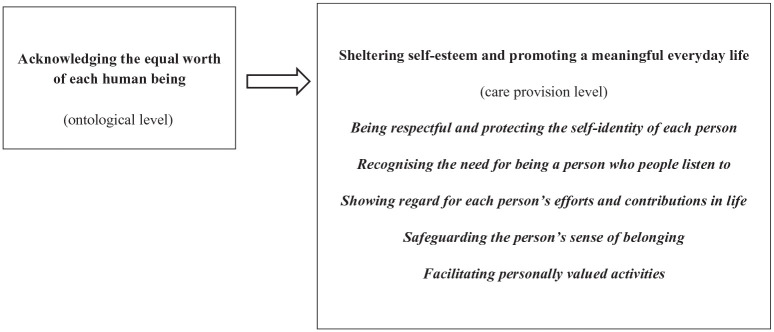
Critical qualities in dignity-preserving care for persons living with dementia in OAHs in Nepal.

Based on our data analysis and interpretations of the HCPs’ perceptions, we identified two themes and five sub-themes as critical qualities inherent in dignity-preserving care for persons with dementia living in OAHs in Nepal. The themes are at two different levels. The first theme ‘Acknowledging the equal worth of each human being’ addresses an ontological care level based on a view of humanity that recognises the inherent dignity of each individual with dementia, by virtue of being human. The second theme ‘Sheltering the self-esteem and promoting a meaningful everyday life’, with five sub-themes, describes a practical care level of how the persons with dementia’s dignity can be preserved in everyday care practise (see Figure 1).

### Theme One: Acknowledging the Equal Worth of Each Human Being

The first theme explores the concept of ‘Acknowledging the equal worth of each human being’. Our analysis indicates that the Nepalese HCPs perceived dignity-preserving care for persons with dementia as anchored in the ontological view that all people are of equal worth and entitled to equal respect.

‘Man-Maryada’ (Dignity in Nepali) means others should not hate or look down upon. I think, we should not hate other people and feel like “I am big and others are small”. We should not have such feelings. We should behave nicely to everyone and give them respect and we are all equal. (Participant #3)

This fundamental belief regarding the inherent dignity of all human beings seemed to lay foundations for HCPs’ daily caregiving attitudes and behaviours, guiding them to treat each person with dementia like other fellow human beings, regardless of their age, cognitive disabilities or health conditions.

I think everyone has equal rights to live as a human being. People with dementia need to be treated in the same way we treat other people. Yah, despite dementia, they need to be treated well. They need to have cleanliness and hygiene. We can’t leave them in a dirty condition. They need good food and proper cleanliness like other people. Even though, they don’t understand, their health needs those things. (Participant #1)

HCPs’ perspectives regarding the equal rights of each individual to basic needs and their worth to receive the same level of treatment and care, including persons with dementia, was found to be foundational towards the HCPs’ dedication to provide cleanliness, hygiene and nutritional needs on a daily basis to the persons with dementia, despite their inabilities to understand and communicate their own needs to the HCPs.

### Theme Two: Sheltering Self-Esteem and Promoting a Meaningful Everyday Life

While the first theme describes the HCPs’ belief in the inherent dignity of all people, asserting that everyone is of equal worth and entitled to equal respect, the second theme outlines how this ontological view was expressed through dignity-preserving attitudes and actions in daily care practice. Our interpretations indicate that this view of humanity served as a foundation that shaped their concrete dignity-preserving attitudes, behaviours and actions in their daily relational interplay with the persons with dementia. In practice, dignity-preserving care was understood to require thoughtful, deliberate attention aimed at protecting the self-esteem of persons with dementia and supporting a meaningful everyday life.

Suddenly, someone here might have a desire to play with a ball or fly a kite, so we provide them these things so that they get involved in something even though for a short period of time. They have a very short span of such demands and don’t care about these things later on. (Participant #4)

HCPs’ perspectives on dignity emphasised the importance of recognising the individuality of each person with dementia and providing them the opportunities to live life with sense of purpose, significance and fulfilments, within the framework of dignity-preserving care. With this belief, HCPs expressed their commitment to acknowledge, prioritise and fulfil the personal needs and desires of the persons with dementia, ensuring that they were valued. This theme comprised five sub-themes that captured how participants enacted dignity-preserving care in daily practice. These sub-themes reflected daily care practices aimed at protecting the self-esteem of persons with dementia and supporting meaningful engagement in everyday life. Each of these care practices are described as sub-themes, below.

#### Sub-Theme One: Being Respectful and Protecting the Self-Identity of Each Person

In the everyday act of living, HCPs described that dignity-preserving care was related to the persons with dementia’s experience of being loved, respected and supported to maintain their self-identity. Among the persons with dementia, the risk of indignity was perceived as related to their experience of cognitive decline and loss of bodily autonomy. These changes were perceived to threaten their own self-image and self-respect, while preserving their body image helped preserve their dignity experience in daily life. Providing assistance with basic needs – such as feeding, personal hygiene, and access to appropriate medical care – was emphasised as a way of demonstrating respect and supporting the preservation of each person’s self-identity, thereby promoting well-being and safeguarding their sense of integrity and dignity.

In our morning care, we change their dress and clean them completely with soap water and cotton scrubs. Then, they are given an oil massage, grooming and finally changing their dresses and combing their hair. For women, we also do some make-up stuff like wearing ‘Tika’ (a red dot worn by married women on their forehead) and nail polish, but only if they want to. If there are clear instructions from the family members or themselves that they do not like that, then we do not apply make-up. (Participant #4)

In situations when facing persons with dementia with challenging illness-related behaviours, such as agitation and aggression, HCPs had focused attention preserving their dignity experience by treating them with politeness, and handling such episodes with respect and patience.

They need care very much. We must speak very softly. The first thing is politeness because Alzheimer’s patients can be very aggressive. We have to deal with this (aggressive behaviour) by talking to them politely. They can start throwing things randomly and turn away when we try to feed them. Sometimes, it takes more than half an hour to feed them. Even if they pass urine, we still must remain very patient. (Participant # 8)

Another aspect perceived as critical for dignity-preserving care was related to respecting persons with dementia’s privacy and being sensitive to their individual needs and preferences.

Regardless of whether they understand or know it or not, we always maintain privacy for them when they are changing clothes because it is their personal right. We always respect their dignity and we try hard to protect their dignity. We always put ourselves in their place. We greet them every day and maintain privacy while changing their clothes. (Participant #4)

The HCPs were attuned to the persons with dementia’s basic needs and simultaneously acted to protect their right to privacy. In everyday care, HCPs’ empathetic understanding of the persons with dementia’s perspective and treating each of them with the same respect and consideration that HCPs would have wanted for themselves were perceived to be a critical quality of dignity-preserving care.

#### Sub-Theme Two: Recognising the Need for Being a Person Who People Listen To

For the HCPs, another important aspect of dignity-preserving care was ensuring that the person with dementia felt seen, heard, and treated as someone whose voice mattered. Besides giving the HCPs access to each person with dementia’s own expressions of needs and concerns, they found that the experience of being recognised as a person through such reciprocal back-and-forth conversations was an important aspect protecting the persons with dementia’s self-esteem. Acknowledging the persons with dementia’s requests and trying to fulfil them was perceived as a vital aspect of respect for their autonomy and their expressed wishes and needs.

We have to politely listen to whatever they tell us. We need to be a good listener. After that, we point out the positive things they have asked us to do for them and we try very hard to accomplish whatever they have asked us to do. Sometimes, they like to go on an outing. At that time, we call their visitors and send them along for an outing. Thus, we try to fulfil as much of their wants and desires as possible. Then, they feel that we respect them. (Participant #4)

The HCPs’ emphasised empathic and active listening in interactions with persons with dementia as they found this to be a core foundation for fostering a respectful, interactive and supportive relationship. The HCPs perceived that, in protecting the right of persons with dementia to have personal authority and autonomy in their own life, recognising their need of being respected partners in dialogue was vital and thus essential in dignity-preserving care.

HCPs often encountered moments when the persons with dementia recalled their memories from the past, particularly from their childhood and youth and expressed the experiences of particular events. In such moments, they often lost awareness of the present time, place, and people, instead becoming immersed in past events and interacting with HCPs as if they were reliving those experiences.

Sometimes, they remember things from their past when they were young, while forgetting the present. For example, when they were married and had small kids playing around. Some say: “It is too late. I need to go to my farm for picking vegetables. Where are our kids? They need to have lunch before I go.” We cannot say directly: “You are right now in this OAH. Now, you are at this age and you remember those things.” Instead, we respond with: “You can keep the foods covered over here so that they will come and eat later. If you are going to pick grass, take this bag to put the grass in.” At least they become happy then. We respond in such a way to give them some consolation. (Participant #2)

In these situations, the HCPs found it dignifying to behave and respond with great sensitivity, avoiding direct contradiction and instead embracing the memories that brought happiness and comfort to the persons with dementia. Showing attentive interests and engaging in these interactions, HCPs not only valued them as fellow human beings but also strengthened their connection to their personal history.

#### Sub-Theme Three: Showing Regard for Each Person’s Efforts and Contributions in Life

During interactions with persons with dementia, HCPs emphasised acknowledging their past efforts and life contributions – such as those made during their working years – as a meaningful way to preserve dignity in care. By acknowledging that individuals with dementia had made important life contributions – even long after the fact and despite cognitive decline – HCPs reinforced their commitment to respectful care, helping to support and strengthen each person’s sense of being appreciated and valued in a vulnerable state.

They are persons with dementia. If you are disrespecting them, their dignity is hurt. Their value will be nothing. But if you provide care with respect and politeness, this will help maintain their dignity because they might have had significant experiences and achievements in their lives. They might have been a police officer, a pilot or held other important roles, but after coming here and after having dementia, if people disrespect them, whatever they did in life, this no longer has any value. (Participant #6)

Recognizing each person with dementia as shaped by their unique background, life history, and profession was seen as key to sustaining relational care and promoting dignity through person-centred practices.

#### Sub-Theme Four: Safeguarding the Person’s Sense of Belonging

While exploring the HCPs’ perspectives regarding dignity in care, we identified their understanding of an integral connection between safeguarding the persons with dementia’s belongingness and preserving their dignity. The HCPs observed that persons with dementia had less contact with their families after moving into an OAH residence. To the HCPs, the persons with dementia’s sense of belongingness were a crucial aspect of dignity-preserving care and, therefore, they aimed to help the persons with dementia to have contact with their family members. While living in the OAH environment, regular family involvement was seen as important for persons with dementia, fostering a sense of togetherness with their loved ones and providing opportunities to celebrate milestones together. The importance of family involvement was emphasised by one participant who explained the role of visits in supporting residents’ well-being.

We have to look after many residents here and one thing that is possible for their family members is to visit them. Sometimes, the family members come here and take them for visits outside for one or two days like during festivals and we like that to happen more often. (Participant # 11)

The HCPs did, however, experience that most persons with dementia tended to feel lonely, and left alone at the OAH by their family. In this context, the risk of their experience of indignity was perceived to stem from a gradual loss of social status as they transitioned into OAHs due to age and dementia. Experiencing being left behind by their families, previous relationships and social networks, weakened their sense of belongingness, and the HCPs saw this as a threat to the persons with dementia’s sense of dignity, which they had to try to alleviate.

In terms of the family, they (persons with dementia) tell us that they feel like their family abandoned them in their time of need. They feel like when they are fit and fine, their family cared for them and now they are disabled so their family abandoned them over here. Then, we tell them that it is for their betterment that their family members have left them over here. We try to console them that in order to take care of them, their children also need to earn a living and earn money as well. We are here as well like their children to take care of them, and when we console them like that, they do tend to understand us. (Participant #11)

In their efforts to safeguard the persons with dementia’s sense of belonging, HCPs also found it vitally important to build close relationships with them, addressing them as ‘father’ and ‘mother’, reflecting the cultural tradition of respecting senior citizens in Nepal.

While we take care of them, like while feeding them, bathing them or changing their clothes, we need to speak to them very politely and treat them well. Saying, “mother, please come and I will help you take a bath”. Or, while feeding them, instead of ordering them like, “Eat this”, we can say, “mother, please eat this”. We need to give them the feeling that they are not alone here and make them feel as though they are connected to us or provide them the sense of being in a family. (Participant #2)

By consciously choosing words and actions that conveyed belongingness and familial relationship, the HCPs aimed to create a homelike and dignity-preserving environment for persons with dementia.

#### Sub-Theme Five: Facilitating Personally Valued Activities

The HCPs did relate dignity-preserving care to being involved in persons with dementia’s everyday life, aiming to make them meaningful while engaging in daily activities. Activities like going to the temple for those OAHs having a temple nearby, attending religious prayers and having the opportunity to express reverence and adoration of your deity were found to be meaningful for persons with dementia who were religiously and spiritually inclined.

The persons with dementia over here who like to pray and worship God are specially bathed by us in the morning, and we give them flowers and worship materials to pray and we also play worship music through the speakers for them. (Participant #7)

Another HCP expressed it like this:

The most important thing is that we need to talk and interact with them and keep them involved. People who can walk are taken to a temple nearby where they can worship God. (Participant #4)

HCP also emphasised engaging the persons with dementia in different leisure pursuits by organising activities of interest like games, exercises and taking them for short walks around the OAHs, as such a state of being active seemed to alleviate their dementia-related challenges and concerns while providing them with a sense of dignity and purpose in everyday life.

Training in activities such as making thread candles and weaving are important. We can also involve them in games such as puzzle games and indoor games. Involving them in such activities means that they don’t have time to think about other things and forget about their issues. (Participant #1)

In addition, HCPs found that organising social activities where all the persons with dementia participated jointly, and were playing music, dancing, watching television or having conversations with each other, for example, was meaningful and dignity-preserving care measures.

There are so many different important things like social interactions. The older mothers and fathers over here feel lonely and they need interaction and they need to be involved in different activities. (Participant #1)

The common group activities were recognised to be significant for the persons with dementia as such acts of socialisation helped them to overcome loneliness by strengthening their experience of being in a social community with others.

## Discussion

Our study identified critical qualities inherent in dignity-preserving care for persons with dementia living in OAHs, as perceived by HCPs caring for them. By exploring HCPs’ beliefs concerning the ontology of care, we discuss how their view of humanity – that each person with dementia has inherent dignity by virtue of being human – shaped their caring attitudes and behaviours towards the persons with dementia. Furthermore, the study examines how this ontological foundation laid the groundwork for their everyday dignity-preserving care practices. This increases our understanding of the critical qualities of dignity-preserving care within the cultural context of Nepal and expands our knowledge of this subject as a global standard of care.

Firstly, the HCPs conceptualised dignity as encompassing respect for each person and a sense of equality among all individuals as human beings – an understanding that can be described as an ontological foundation. A similar finding is described by Dwyer et al. (2009) who found that Swedish nursing home staff expressed that seeing and respecting the resident as the person they are is vital for promoting residents’ dignity. Our finding is also consistent with the Norwegian study of Tranvåg et al. (2015), which found that HCPs who treated residents with dementia as equal human beings helped confirm their experiences of human worth, enhancing their sense of dignity. We here would like to link HCPs’ perspective on dignity to the Canadian social scientist and theorist Nora Jacobson’s concept on *human dignity* in order to create a theoretical framework for our discussion. According to Jacobson (2009), *human dignity* refers to the inherent quality of human beings present in every human by the virtue of being human. Such an intrinsic form of *human dignity* is inalienable – it cannot be created or damaged, irrespective of the contexts or attributes. In line with this understanding of *human dignity*, our finding suggests that the HCPs’ daily care giving attitude and behaviours were based on the foundation that individuals with dementia possess equal rights to the basic needs due to their inherent quality as human beings – worthy of same level of care and treatment as others.

Secondly, on the practice level, the Nepalese HCPs’ perceptions of critical dignity-preserving qualities can be theoretically described in relation to Nora Jacobson’s concept of *social dignity*, which is a form of dignity that is socially created and hence can be measured and preserved but also violated (Jacobson, 2009). Social dignity comprises *dignity-of-self* and *dignity-in-relation. Dignity-of-self* relates to the experience of self-respect and self-worth of an individual. We argue that the Nepalese HCPs’ ways of showing respect and equality towards persons with dementia, for example in assisting them with their daily basic needs to help preserve body image, were aimed at enhancing the persons with dementia’s sense of self and their perceived worth. The HCPs also paid close attention to how the *dignity-of-self* of persons with dementia could be preserved when experiencing illness-related cognitive decline and loss of bodily autonomy. As demonstrated in the Dutch study of Oosterveld-Vlug et al. (2013), by emphasising residents’ interests, past routines and meaning in everyday lives, and paying attention to aspects that characterise the particular resident, HCPs can confirm the self and the identity of residents in the nursing home setting. Similarly, based on our finding, we argue that HCPs’ active listening and acknowledgment of each person with dementia’s life history served as ways of confirming them as valued persons and affirming their *dignity-of-self* – what Jacobson (2009) refers to as *dignity-in-relation*. This aspect pertains to the ways in which respect and worth are conveyed through collective behaviour in the social context and also adheres to social status and rank (Jacobson, 2009). Studies conducted in Norway (Tranvåg et al., 2013, 2015) have shown that relational interactions characterised by HCPs’ humane warmth and understanding where persons with dementia are met as equal human beings and their worthiness and sense of self are affirmed – are essential to preserving dignity. In our study, the Nepalese HCPs emphasised preserving the persons with dementia’s dignity by showing respect and appreciation for their past efforts and contributions in their personal and professional lives. Likewise, valuing them as important family members, supporting their continued social connections with family, friends, and the broader community, and creating opportunities to form new relationships within the OAHs were perceived as vital means for maintaining their sense of dignity – preserving what Jacobson (2009) refers to as the *social dignity.*

Creating a humane and purposeful environment has been found to be a crucial foundation for dignity-preserving care for persons living with dementia (Tranvåg et al., 2013). In their Swedish study, Roos et al. (2023) describe how the experience of dignity and well-being among older persons living in residential care facilities is linked to person-centred care, which supports them in managing daily life, gaining support and influence, and feeling a sense of social belonging. We argue that our findings regarding the HCPs’ perspective in the Nepalese context demonstrate qualities that are both dignifying and person-centred. In the efforts of HCPs to support persons with dementia in managing daily life – through organizing personally meaningful activities, engaging in respectful communication, and fostering a sense of belonging – we recognise person-centred care as a core foundation of their dignity-preserving approach. The pioneer work of British social psychologist and theorist Tom Kitwood, on ‘Positive Person Work’ and ‘personhood’, provides an important theoretical basis for understanding vital dignity-preserving perspectives on the one hand, and ‘malignant social psychology’, on the other – a concept that refers to behaviours which threaten or undermine a person’s dignity (Kitwood, 1993, 1995, 1997; Mitchell & Agnelli, 2015). From our perspective, the concept of ‘personhood’– understood as the inherent value of an individual beyond physical or cognitive abilities (Kitwood, 1997) – aligns closely with what Jacobson (2009) describes as inherent *human dignity*. From their respective perspectives, both concepts serve as important theoretical lenses that help us understand ontology and humanity as the primary foundation for the Nepalese HCPs’ approach to dignity-preserving care. Furthermore, the HCPs’ perception of dignity-preserving attitudes and behaviours – specifically, how respectful and positive interactions contribute to improved experiences and well-being for persons with dementia – can be understood as ‘Positive Person Work’. This concept, as outlined by Kitwood (1997), emphasises recognition, negotiation, collaboration, play, creativity, and facilitation, all of which support the preservation of residents’ *social dignity* (Jacobson, 2009).

The results illuminate several aspects of dignity-preserving care ‘on the practice level’ such as providing assistance in personal hygiene, nutrition and medical care, protecting the right to privacy and autonomy while showing empathy, kindness and politeness during care giving practice. At this point, we will discuss our findings with existing research on dignity-preserving care, highlighting both similarities and differences between the perceptions of Nepalese HCPs and those of HCPs in high-income countries, beginning with the similarities. Recently, Roos et al. (2023) found that the experience of dignity among older persons living in residential care facilities in Sweden was closely linked to the preservation of their identity. This was achieved through the support in managing daily life, opportunities for influence, and a sense of belonging within their social contexts. Likewise, treating residents with respect and safeguarding their privacy, autonomy, integrity and individuality have been identified as the most vital elements of dignity-preserving care in several European countries (Dwyer et al., 2009; Oosterveld-Vlug et al., 2013). By synthesising studies conducted in Australia, England, Sweden and Norway, Tranvåg et al. (2013) found that HCPs viewed compassion for the person with dementia as a fundamental basis for dignity-preserving care. In our study, we identified similar aspects, as Nepalese HCPs perceived active and empathetic listening during interactions with the persons with dementia as vital for dignity-preserving care. This approach created space for them to share their worries, wishes, and needs, as well as to recall meaningful memories from the past. A Scandinavian study by Lohne et al. (2017) found that the dignity experience of nursing home residents were fostered when HCPs actively listened to residents’ life stories, lived experiences, habits, and personal preferences. Similarly, several European studies report that residents with dementia feel more dignified when they are taken seriously and actively listened to by HCPs (Heggestad et al., 2013; Heggestad & Slettebø, 2015; Tranvåg et al., 2015; van Gennip et al., 2016). Likewise, in our study, we found that Nepalese HCPs emphasised the importance of acknowledging the past efforts and contributions of persons with dementia, viewing this recognition as a dignifying aspect of care. Some European studies have previously reported that reflecting on one’s life journey and acknowledging personal achievements – such as honourable employment and the establishment of a family – can evoke feelings of dignity and pride among persons with dementia (Oosterveld-Vlug et al., 2014; Tranvåg et al., 2016).

The Nepalese HCPs also emphasised that sense of belonging – both to family members and to the HCPs within the OAHs – was a critical source of dignity experience for persons with dementia. They noted that the loss of this sense of belonging posed a significant threat to the individuals’ experience of dignity. Previous studies conducted in the United Kingdom, Australia, and Norway (Dening et al., 2013; R. Fleming et al., 2015; Sagbakken et al., 2017) have also recognised that continued engagement and contact with family members is essential in preserving the dignity of nursing home residents. In our study, we found that the HCPs addressed persons with dementia as *father* and *mother* during daily relational interactions – a practice that reflects Nepal’s long-standing cultural tradition of showing respect towards senior citizens. In this regard, HCPs intentionally integrated cultural norms and values by treating persons with dementia as fathers and mothers, drawing from a well-known Sanskrit phrase; ‘Matridevo Bhava’ (regard your mother as a God) and ‘Pitridevo Bhava’ (regard your father as a God), which reflect a long-standing tradition of reverence for seniors in Nepalese society (Chalise et al., 2007). The deliberate use of cultural elements and values has previously been identified as an effective strategy for fostering social inclusion among persons with dementia (Motta-Ochoa et al., 2021). Building on this, we found that the culturally grounded approach of HCPs in our study played a significant role in dignity-preserving care. Through the provision of emotional support and the creation of a homelike environment, HCPs enhanced the sense of belonging among persons with dementia living in OAHs.

The HCPs also emphasised that the dignity of persons with dementia could be preserved by organising individually meaningful activities and group-based recreational pursuits – such as games, exercises, and short walks around the OAHs. These activities were seen as facilitating worthwhile experiences and helping persons with dementia overcome their loneliness and emotional distress. Previous studies from Canada and Norway have also highlighted the importance of providing opportunities for persons with dementia to engage in activities that prevent passivity and they are personally meaningful. Such activities stimulate positive emotions, cognitive function, and engagement (Roland & Chappell, 2015; Tranvåg et al., 2016), and are considered crucial components of dignity-preserving care for people living with dementia (Tranvåg et al., 2016).

The Nepalese HCPs also emphasised that the dignity of persons with dementia could be preserved by facilitating their participation in religious and spiritual activities. Practices such as accompanying spiritually devoted persons to nearby temples, playing religious music, and singing prayers as part of daily routines were perceived as critical qualities of dignity-preserving care. S. Shrestha and Zarit (2012) previously identified religious and spiritual faith as an important aspect contributing to the quality of life among women residing in OAHs in Nepal. Residents who engaged in religious and spiritual activities and held a belief in God were found to experience relief from their worries and demonstrated better coping with life’s difficulties.

Previous studies conducted in several European countries and the United States have indicated that spiritual engagement – which encompasses experiences of transcendence and spiritual meaning, the preservation of religious beliefs and personal values, feelings of closeness to God, and a deep sense of connectedness with nature – can strengthen the sense of dignity among persons with dementia (R. Fleming et al., 2015; Nåden et al., 2013; Oosterveld-Vlug et al., 2014; Palmer, 2013; Tranvåg et al., 2016; Van der Geugten & Goossensen, 2020). Similarly, in the present study, spiritual care was found to foster a sense of contentment in daily lives of persons with religious beliefs. Integrating spirituality into dementia care was perceived as a way of reconnecting with the core, dignifying values that had shaped these persons lives.

Having discussed the similarities with findings from high-income countries, we now explore the important differences that warrant attention. In the present study, Nepalese HCPs appeared to place greater emphasis on fostering a sense of belonging among persons with dementia than has been documented in previous research. This is exemplified by culturally rooted practices such as referring to them as ‘mother’ and ‘father’ – a traditional expression of respect for senior citizens – and by efforts to create a home-like environment that nurtures emotional and social connections. The emphasis on spiritual care – particularly in ensuring that the religious needs of persons with dementia are met – also appears more pronounced in the perspectives of Nepalese HCPs than in previous research. As noted in the preceding paragraph, this highlights a culturally embedded dimension of dignity-preserving care that may be less emphasised in other contexts.

## Implication for Practice

In its *Global action plan on the public health response to dementia* 2017–2025, World Health Organization (2017) emphasises the importance of providing people with dementia the care and support they need to live with dignity. Conducting research on dignity-preserving care in regions underrepresented in the gerontological nursing literature is therefore essential. This study contributes new knowledge about the critical qualities of dignity-preserving care in a context that has previously received limited attention. Our findings highlight how Nepalese HCPs in OAHs perceive and provide dignity-preserving care, rooted in their local socio-cultural values and practices. These findings can inform the strategic planning of not only Nepalese OAHs management but may also inform practice in similar low- and middle-income countries (LMICs) with comparable institutional and socio-cultural contexts in promoting dignity-preserving care for persons with dementia. By engaging with these insights, HCPs in OAHs and similar institutional settings in Nepal and other LMICs, can deepen their understanding of the essential qualities that underpin such care. In line with Wang et al. (2018), we argue that learning from experiences of HCPs is essential for the development of policies and educational programmes aimed at improving healthcare services for people with dementia. As highlighted by Pathak and Gaire (2020), we also consider it vital that Nepalese HCPs – like their counterparts in other countries and cultures – receive adequate training to provide high-quality dementia care. In such educational programmes, Simkhada et al. (2024) emphasise the importance of providing culturally appropriate learning materials and dementia training tailored to Nepal’s healthcare system – an approach that may be applicable to other LMICs with similar cultural and structural characteristics. We also strongly recommend that authorities and policymakers in LMICS – ensure that the essential critical qualities of dignity-preserving care are given a central place in the national dementia-specific educational programme.

According to the *Global action plan on the public health response to dementia* 2017–2025 (World Health Organization, 2017), all member states are called upon to develop and implement national dementia policies, legislation, strategies, and plans. In this critical effort, we strongly recommend that the Nepalese authorities and policymakers incorporate the critical qualities of dignity-preserving care as a foundational element in national dementia care strategies. Emphasis should be placed on expanding knowledge about this core of care. Furthermore, future research should explore whether healthcare professionals’ sex and gender influence their understanding and practice of dignity-preserving dementia care within the Nepalese context. The experiences of critical qualities of dignity-preserving care, as perceived by persons with dementia in Nepalese OAHs, have not yet been explored. Voicing these persons with dementia in future research could provide a deeper understanding of their lived experiences and perspective of the essence of dignifying care, with possible implications to the similar care settings in other LMICs.

## Strengths and Limitations

We have placed particular emphasis on proving a transparent account of the research process, as advocated by Hiles (2008). Consistent with the hermeneutical method outlined by V. Fleming et al. (2003), we focused on establishing the trustworthiness of the study by applying the quality criteria proposed by Lincoln and Guba (1985) and further elaborated by Guba and Lincoln (1994).

To our knowledge, this study is the first to explore and describe the critical qualities inherent in dignity-preserving care for persons with dementia living in OAHs in Nepal. By exploring the perspectives of HCPs in this region, our study reveals culturally embedded practice and a pronounced emphasis on spirituality in dignity-preserving dementia care – elements that appear to be particularly significant within the Nepalese context. By comparing the findings with existing literature from high-income countries, this study offers new insights into both context-specific and global dimension of dignity-preserving dementia care. This comparative approach contributes to a deeper cross-cultural understanding within dementia care research. However, the study has several limitations. Only 11 HCPs participated, and for seven of them, only a single interview was conducted. A larger sample, along with conducting two or three interviews with each participant, would likely have yielded richer empirical data, particularly given that understanding is anchored in specific historical and contextual situations (V. Fleming et al., 2003). Nevertheless, as Brinkmann (2012) describes, a sample of this size can still generate valuable insights into phenomena about which we have limited prior knowledge. In four of the participating OAHs, persons with dementia had been formally diagnosed by specialists. In the government-run OAH, many residents were believed by HCPs to exhibit dementia-related symptoms, although they had not received a formal diagnosis from specialists. Therefore, we acknowledge the lack of formal diagnosis as a limitation of this study, as it is possible that some residents believed to exhibit dementia-related symptoms may have been affected by other medical conditions. The majority of participants were female, which may have influenced their perceptions of dignity-preserving care. However, potential sex and gender differences in the understanding of such care were not explored in this study.

## Conclusion

This study explored healthcare professionals’ perspectives on dignity-preserving care for persons with dementia in Nepalese older age homes. The findings underscore that healthcare professionals’ views on humanity, cultural traditions, and ethical commitments play a pivotal role in shaping their daily care practices. While this study addresses the crucial issue of care for one of the most vulnerable population in an underrepresented region of the world, it also identifies several commonalities in healthcare professionals’ perceptions of dignity-preserving care – such as respect, autonomy, individuality, and privacy – when compared to findings from high-income countries. However, we also found that culturally influenced tradition shaped Nepalese healthcare professionals’ approach to care, with a stronger emphasis on fostering a sense of belonging, a deeply rooted respect for seniors, and a pronounced spiritual dimension in caregiving. These findings offer research-based insights to support healthcare professionals, healthcare institutions, and policymakers in Nepal in better understanding and implementing dignity-preserving dementia care. We strongly recommend integrating these culturally embedded practices into national policies and training programs, while also incorporating relevant practices from high-income countries through education and research-based interventions. Furthermore, we advocate for increased research in this area – particularly studies that centre the perspectives of persons with dementia – to support the development of dignity-based dementia care models in this region.

## Supplemental Material

sj-doc-1-gqn-10.1177_23333936251369444 – Supplemental material for Dignity-Preserving Dementia Care in Old Age Homes in Nepal: Healthcare Professionals’ Perspectives Supplemental material, sj-doc-1-gqn-10.1177_23333936251369444 for Dignity-Preserving Dementia Care in Old Age Homes in Nepal: Healthcare Professionals’ Perspectives by Oscar Tranvåg and Soni Shrestha in Global Qualitative Nursing Research
