# Supplementary material for: Dignity-Preserving Dementia Care in Old Age Homes in Nepal: Healthcare Professionals’ Perspectives
Source: Glob Qual Nurs Res. 2025 Aug 27;12:23333936251369444. doi: 10.1177/23333936251369444 (PMC12391714; doi:10.1177/23333936251369444)
Supplement: sj-doc-1-gqn-10.1177_23333936251369444 – Supplemental material for Dignity-Preserving Dementia Care in Old Age Homes in Nepal: Healthcare Professionals’ Perspectives [file sj-doc-1-gqn-10.1177_23333936251369444.doc]

**GUIDE FOR DIALOGUE WITH THE STUDY PARTICIPANTS**

1. Can you tell me little about your educational and professional background?

- *What is your educational background?*
- *How long have you been working at this elderly homes?*

2. How will you describe the care/ caregiving work you have been providing for residents with dementia while working at the old age home?

3. How do you understand the terms ‘dignity’ and dignity-preserving care?

- *What does dignity mean to you?*
- *What does dignity-preserving care mean to you as a healthcare professional caring for persons with dementia living at the old age home?*

4. Can you tell me how the dignity of residents with dementia can be preserved, as you perceive this from your professional perspective?

- *Based on your experience and perception, what does dignity mean for residents with dementia?*
- *What are the critical qualities of dignity-preserving care?*
- *Can you share an experience where you felt that dignity of the persons with dementia was preserved during your care practices?*

5. How does residents’ dignity get violated?

- *What can contribute to violating the residents' dignity? Can you share an experience where you felt that dignity of the persons with dementia was preserved during your care practices?*
- *How do you handle those situations?*
- *How can such violations be avoided?*

6. Finally, is there anything you would like to add – concerning dignity and dignity-preserving care for residents with dementia?
